# TIMP-1 and VEGF-165 serum concentration during first-line therapy of ovarian cancer patients

**DOI:** 10.1186/1471-2407-10-139

**Published:** 2010-04-13

**Authors:** Sven Mahner, Linn Woelber, Christine Eulenburg, Joerg Schwarz, Walter Carney, Fritz Jaenicke, Karin Milde-Langosch, Volkmar Mueller

**Affiliations:** 1 Department of Gynecology and Gynecologic Oncology, University Medical Center Hamburg-Eppendorf, Hamburg, Germany; 2 Department of Medical Biometry and Epidemiology, University Medical Center Hamburg-Eppendorf, Hamburg, Germany; 3 Oncogene Science/Siemens Health Care DX, Cambridge, MA, USA; 4 Department of Gynecology, Asklepios Klinik Nord - Heidberg, Hamburg, Germany

## Abstract

**Background:**

Angiogenesis appears to play an important role in ovarian cancer. Vascular endothelial growth factor (VEGF) has recently been implicated as a therapeutic target in ovarian cancer. The tissue inhibitor of metalloproteinase 1 (TIMP-1) is involved in tissue invasion and angiogenesis. The application of serum TIMP-1 and VEGF to monitor primary therapy and predict clinical outcome of patients with ovarian cancer is unclear.

**Methods:**

Patients with epithelial ovarian cancer who presented for primary surgery were included in this study. A total of 148 serum samples from 37 patients were analyzed. Samples were prospectively collected at 4 predefined time points: 1. before radical debulking surgery, 2. after surgery and before platinum/taxane based chemotherapy, 3. during chemotherapy, 4. after chemotherapy. Serum VEGF-165 and TIMP-1 as well as CA-125 were quantified by ELISA or ECLIA and correlation with response and long-term clinical outcome was analyzed.

**Results:**

Serum levels of all markers changed substantially during first-line therapy. High CA-125 (p = 0.002), TIMP-1 (p = 0.007) and VEGF-165 (p = 0.02) after chemotherapy were associated with reduced overall survival. In addition, elevated CA-125 (p < 0.001) and VEGF-165 (p = 0.006) at this time point predicted poor progression-free survival. TIMP-1 and VEGF-165 were closely correlated at all time-points during therapy.

**Conclusions:**

TIMP-1 and VEGF serum levels changed significantly during first-line therapy of ovarian cancer patients and predicted prognosis. These findings support the role of angiogenesis in ovarian cancer progression and the use of antiangiogenic therapy.

## Background

Epithelial ovarian cancer accounts for the highest mortality among all gynecologic malignancies in the Western world. The American Cancer Society estimates more than 20.000 new cases and 15.000 deaths due to this disease each year in the United States [1]. Because of the absence of specific early symptoms most patients are diagnosed at an advanced stage with extensive intra-abdominal disease and peritoneal carcinomatosis. Initial treatment for ovarian cancer consists of aggressive surgical cytoreduction and six cycles of carboplatin-paclitaxel combination chemotherapy [2]. Most patients will achieve radiologic and serologic complete remission after first-line therapy. However, 75% of these patients will relapse and eventually die of their disease [3]. Clinical trials of the past decade have mainly focused on adding cytostatic agents to the established combination of carboplatin and paclitaxel to improve therapy. Unfortunately, this approach provided no benefit and development of new therapeutic strategies is required [4].

Enhanced understanding of the underlying biology of ovarian cancer has recently led to the discovery of molecular targeted therapies to accompany chemotherapy and potentially improve outcome [5]. Currently, angiogenesis appears to be the most promising therapeutic target for ovarian cancer [6-9] and large phase III trials with anti-angiogenic therapeutics are conducted worldwide. In this context, the development of predictive and prognostic markers to optimize the use of these targeted approaches in clinical practice is one of the most important issues. Established prognostic factors in patients with epithelial ovarian cancer are International Federation of Gynecology and Obstetrics (FIGO) stage, residual tumor volume after primary surgical cytoreduction, tumor grading and histological subtype [10]. Several other clinical and biological factors including serum cancer antigen 125 (CA-125) have been assessed for prognostic and predictive relevance [11,12].

CA-125 is a high molecular weight glycosylated membrane protein that can be detected in serum and is elevated in more than 80% of patients with ovarian cancer. It is used as a marker to assess therapy-response and progression [13,14]. A rapid decrease of CA-125 during chemotherapy predicts a favourable prognosis and might also serve as a marker for disease recurrence. However, 20% of ovarian cancers have low or no expression of CA-125, thus, additional serum markers are required.

Degradation of basement membranes and extracellular matrix as well as neovascularisation are essential features in the progress of ovarian cancer. Vascular endothelial growth factor (VEGF) and tissue inhibitor of metalloproteinase-1 (TIMP-1) are involved in these processes and might therefore serve as potential biomarkers in ovarian cancer patients [15,16].

The aim of the current study was to evaluate the role of serum VEGF-165 and TIMP-1 to monitor therapy and predict clinical outcome in patients with ovarian cancer in comparison to established clinicopathological parameters and CA-125.

## Methods

### Patients

Patients with epithelial ovarian cancer who presented for primary surgery at the University Medical Center Hamburg-Eppendorf between 1996 and 2004 were included in this study. A total of 148 serum samples from 37 patients were analyzed. Detailed patient characteristics are listed in Table 1. Clinicopathologic factors were evaluated by reviewing medical charts and pathological records. Tissue slides were reviewed for histological classification and clinical outcome was followed from the date of surgery to the date of death or until the end of 2007. Response to therapy was evaluated with the biomarker CA-125. All patients gave written informed consent to access their tissue/serum and to review their medical records according to our investigational review board and ethics committee guidelines. The use of medical records, serum and tumor tissue was approved by the ethics committee of the Medical Board Hamburg (reference number #190504).

**Table 1:** Patient characteristics

No. of patients	37
Age (years)	

mean	58

median	61

range	26-78

**FIGO-Stage**	

I	1

II	1

III	29

IV	6

**Histologic subtype**	

serous	31

endometriod	1

clear cell	1

undifferentiated	4

**Grading**	

1	0

2	9

3	27

not determined/unknown	1

**Lymph node metastasis**	

pN0	11

pN1	18

NX	8

**Perioperative Ascites**	

<500 ml	15

>500 ml	21

not determined	1

**Residual tumor after surgery**	

microscopic	22

macroscopic	14

not determined	1

**Survival (months)**	

progression-free survival	

mean	20

median	16

range	2-71

overall survival	

mean	36

median	32

range	8-98

## Serum samples

Serum samples were collected at 4 predefined time-points: 1. before surgery (within 7 days before surgery); 2. after surgery (within 7 days after surgery) and before chemotherapy; 3. during chemotherapy (within 3 weeks of the third cycle of chemotherapy); 4. after chemotherapy (during the first follow-up visit 3 months after the last cycle of chemotherapy).

After centrifugation at 3400 rpm for 10 min, the supernatants were stored at -20°C before use.

### Quantitative analysis of serum VEGF-165 and TIMP-1 levels

Serum VEGF-165 and TIMP-1 were quantified by commercially available ELISA (Siemens Healthcare Diagnostics, Tarrytown, USA). The serum samples and controls were diluted 1:10 (VEGF-165) and 1:50 (TIMP-1) with sample diluent buffer (containing bovine serum albumin, buffer salts and 0.09% sodium acide). One hundred microliters of the standards, diluted control samples and diluted serum samples were dispensed into 96-well plates (coated with an anti-human monoclonal antibody) and incubated for 1.5 hours at 37°C (VEGF-165) or 30 minutes at room temperature (TIMP-1). Wells were washed and one hundred microliters of the detection antibody (containing biotinylated anti-VEGF-165 monoclonal antibody or alkaline phosphatase-labeled anti-TIMP-1 antibody, respectively) were added.

VEGF-165 plates were incubated for one hour at 37°C, washed and further incubated with one hundred microliters of streptavidin alkaline-phosphatase-labeled conjugate for one hour at room temperature. After washing, one hundred microliters of chromogenic substrate (BluePhos substrate) were added for 45 minutes at room temperature. The reaction was stopped with one hundred microliters EDTA-stop solution and absorbance was read at 650 nm by an automated plate-reader (Tecan, Crailsheim, Germany).

TIMP-1 plates were incubated for 30 minutes at room temperature. After washing, one hundred microliters of chromogenic pNPP-substrate was added for 25 minutes at room temperature in the dark. The reaction was stopped with one hundred microliters of EDTA-stop solution and absorbance was read at 405 nm by automated plate-reader (Tecan, Crailsheim, Germany).

The VEGF-165 and TIMP-1 concentration was estimated from the standard curve. Each sample, standard and control were analyzed in duplicate.

### Quantitative analysis of CA-125

CA-125 serum levels were quantified using the second-generation electrochemiluminescence immunoassay (ECLIA) and the Roche Modular Analytics E170 system (Elecsys module, Roche Diagnostics, Mannheim, Germany) with 20 μl serum samples.

### Statistical analysis

All statistical analyses were conducted using SPSS software version 15.0 (SPSS Inc., Chicago, IL, USA). Changes in the serum level of each marker during therapy were calculated separately using the Wilcoxon test comparing paired samples at successive time points for individual patients.

Correlations between serum CA-125, VEGF-165 and TIMP-1 concentrations and tumor characteristics were calculated by Chi-square tests. For this purpose, cases were divided into two groups of equal size for each marker at each time-point using the median value as cut-off. The resulting groups were compared regarding the following parameters: age: <61 years versus 61 years and older; ascites: <500 ml versus >500 ml; residual tumor after surgery: microscopic versus macroscopic; lymph node status: no metastasis versus lymph-node metastasis; grading: G2 versus G3. Correlations between serum VEGF-165 and TIMP-1 concentrations and treatment response according to CA-125 decrease were evaluated by pair wise Pearson-correlations, assessing the coherence of continuous serum values of VEGF-165 and TIMP-1 and the relative decrease of CA-125 during therapy.

Kaplan-Meier analysis of progression-free survival (PFS) and overall survival (OS) was performed using the same groups. Survival probabilities were compared with the log-rank test. All tests were performed at a significance level of p = 0.05.

## Results

### Patients

A total of 37 patients were included in this study; detailed characteristics are listed in Table 1. All patients underwent radical surgery including hysterectomy, bilateral salpingo-oophorectomy, appendectomy, infragastric omentectomy and systematic pelvic and paraaortic lymphadenectomy as well as resection of all visible tumor. In the majority of patients, complete debulking could be achieved (22 patients with microscopic residual tumor). All patients received platinum based first-line combination-chemotherapy. According to a decrease in CA-125 serum concentrations, all patients responded to first-line treatment except for one (median decrease in CA-125 was 95% (range 54%-100%). Median follow up time was 29 months. In the study cohort, progression free survival ranged between 2 and 71 months with a median of 17 months; median overall survival was 44 months and ranged from 6-97 months.

### Serum concentration of CA-125, TIMP-1 and VEGF-165 duringfirst-line therapy

**Figure 1:**
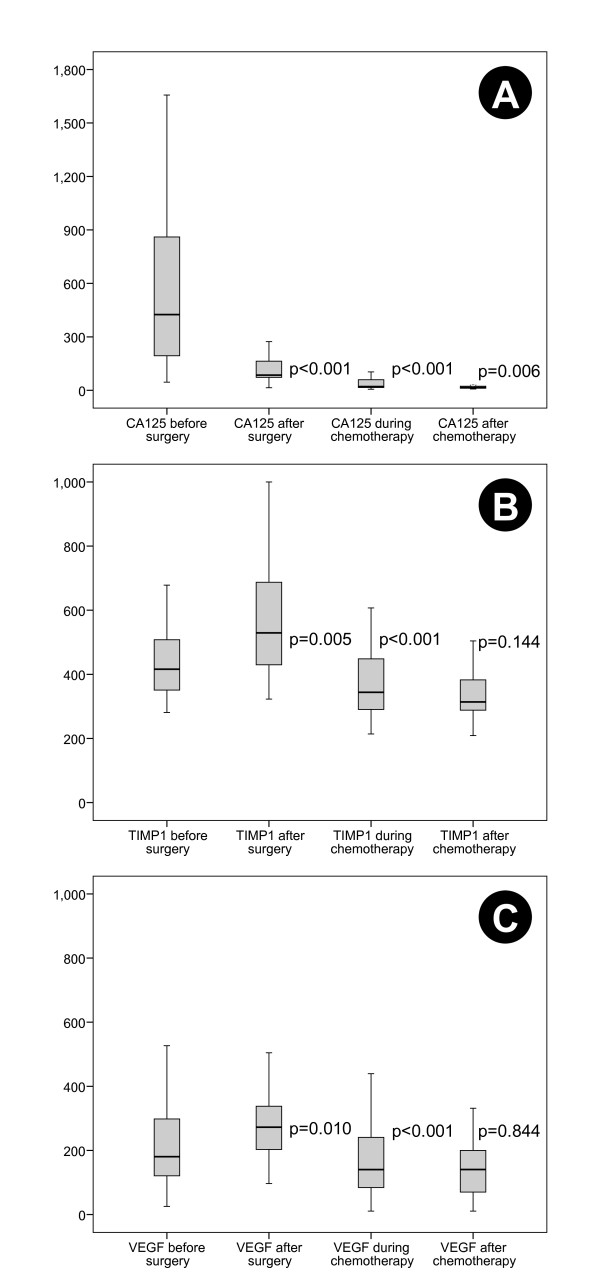
**CA-125 [kU/L] (A), TIMP-1 [ng/L] (B) and VEGF [pg/L] (C) before and after surgery (=before chemotherapy) and during and after chemotherapy (Wilcoxon Tests comparing paired samples at successive time points for individual patients)**.

**Figure 2:**
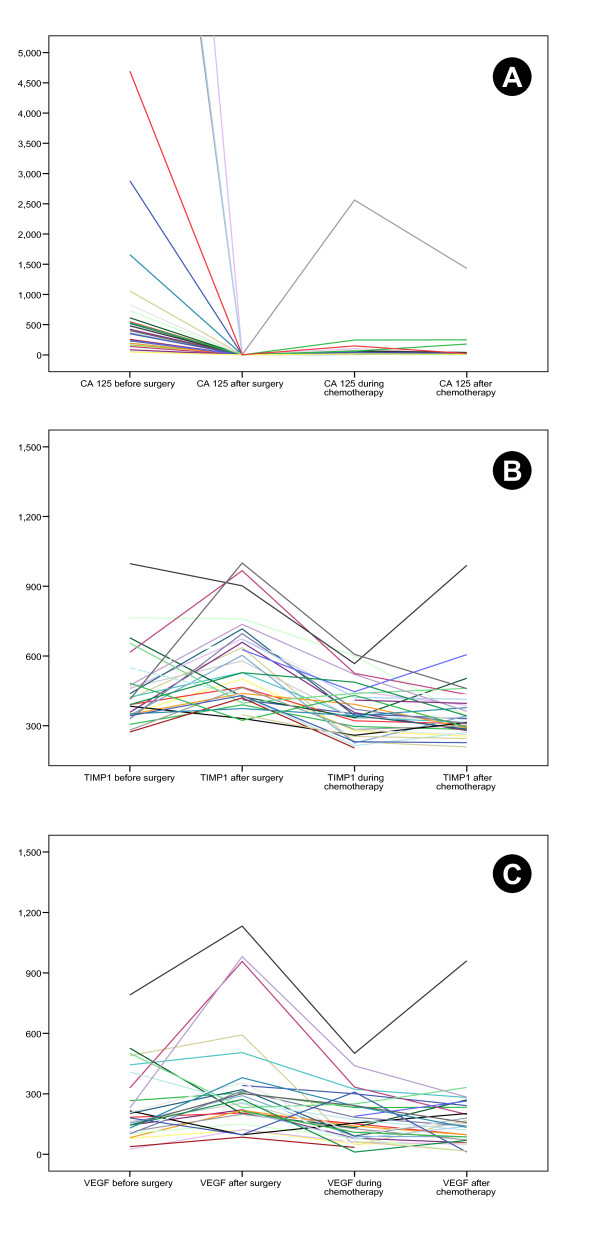
**CA-125 [kU/L] (A), TIMP-1 [ng/L] (B) and VEGF [pg/L] (C) before and after surgery (=before chemotherapy) and during and after chemotherapy presented as linked data for each patient**.

Serum concentrations of CA-125, VEGF-165 and TIMP-1 at the respective time-points are listed in Table 2. CA-125 decreased significantly after surgery whereas the serum concentrations of TIMP-1 and VEGF-165 increased (Figure 1). During first-line chemotherapy the serum concentrations of CA-125, TIMP-1 and VEGF-165 decreased significantly compared to the previous time-point. Further significant decrease after the end of chemotherapy was only observed for CA-125, while TIMP-1 and VEGF-165 remained unchanged. Compared to initial serum levels before surgery, CA-125, TIMP-1 and VEGF-165 during and after chemotherapy also decreased significantly (during chemotherapy: p < 0.001, p = 0.002, p = 0.026 and after Chemotherapy: p < 0.001, p < 0.001, p = 0.001, respectively). Linked data for the different markers in each patient are presented in Figure 2.

**Table 2:** Serum concentrations of CA-125, TIMP-1 and VEGF at different time-points during therapy

	Before surgery			After surgery/before CTX			during CTX			after CTX		
	
	mean	median	range	mean	median	range	mean	median	range	mean	median	range
CA-125 [kU/L]	1948	413	20-20880	325	84	15-5965	113	21	6-2562	72	15	5-1429

TIMP-1 [ng/mL]	451	403	273-887	573	529	323-1000	377	351	204-616	371	333	209-990

VEGF [pg/mL]	231	171	25-791	355	272	85-1133	176	139	11-558	192	147	11-960

### Correlation between serum concentration of CA-125, TIMP-1 and VEGF-165 and clinicopathological factors, response, progression and survival

The serum values of each marker were divided in two groups (high and low) at each time-point using the median as cut-off value. Correlations between CA-125, TIMP-1 and VEGF-165 and progression and survival are listed in Table 3. Correlations between serum markers and clinicopathological parameters are listed in Table 4. Correlations between serum VEGF-165 and TIMP-1 concentrations and treatment response according to CA-125 are shown in Table 5.

**Table 3:** Correlations between serum concentration of CA-125, TIMP-1 and VEGF and survival

	PFS		OS	
	
	p-value	+/-	p-value	+/-
CA-125 before surgery	0.593	.	0.629	.

CA-125 before CTX	0.725	.	0.033*	**-**

CA-125 during CTX	0.627	.	0.308	.

CA-125 after CTX	**<0.001**	**+**	0.002*	**+**

TIMP-1 before surgery	0.534	.	0.195	.

TIMP-1 before CTX	0.499	.	0.980	.

TIMP-1 during CTX	0.781	.	0.380	.

TIMP-1 after CTX	0.152	.	**0.007**	**+**

VEGF before surgery	0.352	.	0.302	.

VEGF before CTX	**0.043**	**+**	0.230	.

VEGF during CTX	0.946	.	0.496	.

VEGF after CTX	**0.006**	**+**	**0.023**	**+**

Correlations between serum concentration of CA-125, TIMP-1 and VEGF (below/above median) and survival. For progression-free survival (PFS) and overall survival (OS): p-values from the log-rank-test. +/- indicates the direction of a significant correlation. A positive correlation indicates a higher likelihood of an event (e.g. elevated CA-125 is associated with high likelihood of disease progression).

**Table 4:** Correlations between serum concentration of CA-125, TIMP-1 and VEGF and clinicopathological factors

	Age		Residual Tumor		Ascites		pN		Grading	
	
	p-value	+/-	p-value	+/-	p-value	+/-	p-value	+/-	p-value	+/-
CA-125 before surgery	**0.046**	**+**	0.631	.	**0.004**	**+**	0.254*		0.121*	

CA-125 before CTX	0.169	.	0.619	.	0.619	.	1.000*		0.118*	

CA-125 during CTX	0.238	.	0.533	.	0.774	.	0.440*		1.000*	

CA-125 after CTX	0.300	.	0.948	.	0.275	.	0.339*		0.238*	

TIMP-1 before surgery	0.273	.	0.812	.	0.096	.	0.423*		0.682*	

TIMP-1 before CTX	0.387	.	0.169	.	0.430	.	0.414*		1.000*	

TIMP-1 during CTX	0.229	.	0.934	.	0.201	.	0.257*		0.429*	

TIMP-1 after CTX	0.086	.	0.157*	.	0.032*	**+**	0.218*		0.688*	

VEGF before surgery	0.273	.	0.597	.	0.550	.	1.000*		0.08*	

VEGF before CTX	0.221	.	0.280	.	0.946	.	0.226*		1.000*	

VEGF during CTX	0.395	.	0.724	.	0.486	.	1.000		0.688*	

VEGF after CTX	0.303	.	0.829	.	0.829	.	0.110*		0.688*	

Correlations between serum concentration of CA-125, TIMP-1 and VEGF (below/above median) and clinicopathological factors. A positive correlation indicates a higher likelihood of an event. P-values from the Pearson-Chi^2^-Test and from Fisher's exact test (*) are reported for binary correlations with age: <61 years versus 61 years or older; ascites: <500 ml versus >500 ml; residual tumor after surgery: microscopic versus macroscopic; lymph node status: no metastasis versus lymph-node metastasis; grading: G2 versus G3. +/- indicates the direction of a significant correlation.

**Table 5:** Correlations between serum concentrations of VEGF-165 and TIMP-1 and response to first-line treatment according to decrease of CA-125.

	Response (% decrease of CA-125)	
	
	p-value	correlation coefficient r
TIMP-1 before surgery	0.375	0.174

TIMP-1 before CTX	0.150	0.265

TIMP-1 during CTX	0.480	0.127

TIMP-1 after CTX	0.554	0.107

VEGF before surgery	0.725	-0.07

VEGF before CTX	0.729	0.065

VEGF during CTX	0.414	-0.147

VEGF after CTX	0.671	-0.077

P-values according to the Pearson correlation coefficients using continous serum values and relative decrease of CA-125.

**Figure 3:**
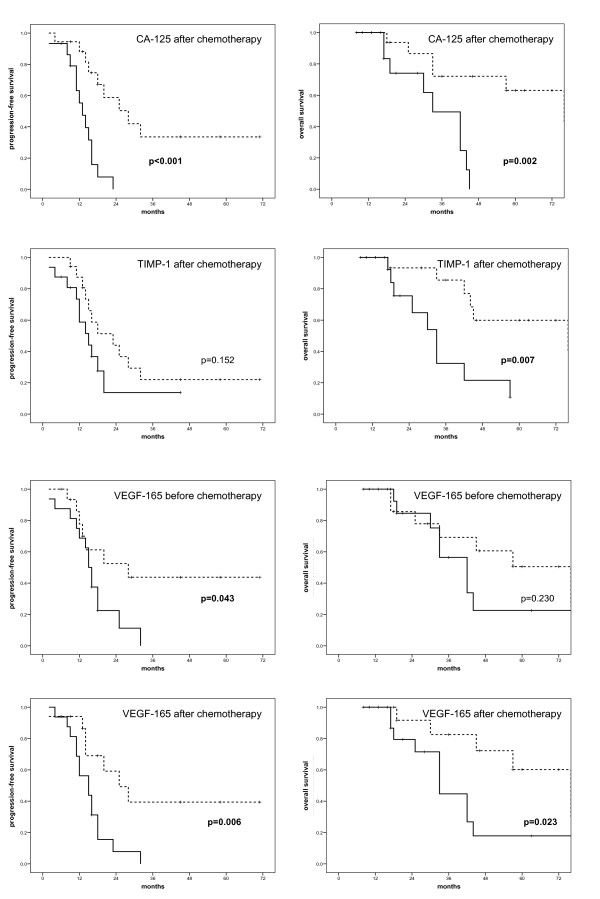
**Kaplan-Meier plots of progression-free survival (left) and overall survival (right) for serum concentrations of CA-125 after chemotherapy, TIMP-1 after chemotherapy, VEGF-165 before chemotherapy and VEGF-165 after chemotherapy**. Dotted line: below median; solid-line: above median.

High serum concentrations of CA-125 before surgery were significantly associated with age (p = 0.046) and large ascites volume (p = 0.004). Increased CA-125 after debulking surgery was associated with improved overall survival (p = 0.033) and low CA-125 after chemotherapy was associated with improved progression-free (p < 0.001) and overall survival (p = 0.002, Figure 3).

High TIMP-1 concentration after chemotherapy was associated with decreased overall survival (p = 0.007) and patients with large ascites volume had higher TIMP-1 concentrations at this time point (p = 0.032).

High VEGF-165 before (p = 0.043) as well as after chemotherapy (p = 0.006) was associated with decreased progression-free survival, while high serum-concentration of VEGF-165 after chemotherapy was also associated with decreased overall survival (p = 0.02).

CA-125 concentration before surgery was inversely correlated with VEGF-165 concentration before surgery (r = -0.460; p = 0.01). No inter-marker-correlation was observed for the other factors except for a constant correlation between VEGF-165 and TIMP-1 before surgery (r = 0.467; p = 0.008), after surgery (r = 0.522; p = 0.002), during chemotherapy (r = 0.437; p = 0.009) and after chemotherapy (r = 0.408; p = 0.017).

## Discussion

To investigate the role of new serologic biomarkers in therapy monitoring and to determine their potential prognostic significance in ovarian cancer, TIMP-1, VEGF-165 and CA-125 were analyzed in 148 serum samples from 37 patients with epithelial ovarian cancer. We could demonstrate for the first-time that changes of VEGF-165 and TIMP-1 during first-line therapy of ovarian cancer are substantial and closely correlated. In addition, we found a potential prognostic and predictive role for TIMP-1 and VEGF-165.

Biomarkers play an important role in the management of ovarian cancer in several aspects. These include monitoring response to treatment, estimating prognosis and predicting response to specific drugs. Of all serologic markers, CA-125 is best established in ovarian cancer. In the follow-up of patients, rising serum concentrations correlate with disease progression in approximately 90% of cases [14]. It can also be used as a surrogate marker for response to chemotherapy, as a decrease in CA-125 by 50-75% correlated with response in several trials [17]. In the current study, we used CA-125 as 'benchmark biomarker' for comparison with new potential markers. As expected, CA-125 levels were high before surgery and decreased substantially during first-line therapy (Table 2, Figure 1). It was significantly associated with ascites volume (p = 0.004, Table 3), a correlation that has previously been described for ovarian cancer as well as other malignancies [18]. We observed no correlation between preoperative CA-125 and survival but a weak, yet significant positive correlation between elevated pre-chemotherapeutic CA-125 and overall survival (p = 0.033). This correlation might just reflect the controversial role of pre-treatment CA-125 in ovarian cancer: Some studies found a prognostic relevance while others failed to demonstrate this impact [11,19,20]. Due to these inconclusive findings, pre-treatment CA-125 has currently no clinical role before initiation of first-line therapy. Patients with low serum CA-125 at the end of first-line therapy had a favourable progression-free (p < 0.001) and overall survival (p = 0.002, Figure 3). Our results are consistent with other studies and confirm the utility of CA-125 as marker for ovarian cancer surveillance [11,21]. However, CA-125 has several limitations. Up to 20% of ovarian cancers are CA-125 negative despite substantial tumor burden [22]. In addition, more than 50% of patients with normalized CA-125 at the end of therapy still have persistent disease [23]. Finally, rising CA-125 does not always precede disease recurrence, thus, additional serum markers are required.

Angiogenesis has been established as a crucial feature of tumor development, growth and spread [24]. VEGF is the most prominent angiogenic molecule and has been shown to parallel tumor growth and metastasis in various cancer types [25] and is currently the most promising target for anti-angiogenic therapy in ovarian cancer [6-8]. In contrast to other targeted therapies like trastuzumab for breast cancer [26], no predictive marker has been established so far for bevacizumab treatment. VEGF could play a role in this situation. Although there is abundant evidence that VEGF plays a central role in the development and growth of ovarian cancer, information regarding the clinical utility of serum VEGF levels is limited and inconclusive. VEGF has been detected in tissue and serum of patients with ovarian cancer [27-33]. It was shown that the serum levels of VEGF are significantly elevated in patients with epithelial ovarian cancer compared to levels in patients with benign disease [33]. In the largest published analysis of 314 patients with ovarian cancer, high preoperative VEGF serum concentrations were associated with decreased overall survival by multivariate analysis [31]. Several other studies produced conflicting results regarding the prognostic impact of serum VEGF [27,28,30,32,34-40]. However, the variation of serum VEGF values in the course of treatment has not been studied since many of these studies used 'pre-treatment' samples and did not separate between pre- and postoperative serum samples or clearly define time points of serum collection.

When comparing different study results there is a high variability of VEGF serum levels, which might be caused by differences in assay techniques (many assays have no specificity for different VEGF-isoforms), storage of specimens and the small number of samples analyzed in most studies. Furthermore, there is no clearly defined 'cut-off' to classify serum VEGF concentrations as elevated or normal. This complicates cross trial comparison and was our rationale to use median values as 'cut-off' for the analyses. With this approach, pre- or postoperative VEGF-165 had no prognostic significance. In the current study, we observed a significant increase between pre- and postoperative serum VEGF-165 levels (p = 0.01, Figure 1, Table 2). Moreover, we found a significant consecutive decrease of serum VEGF-165 during chemotherapy (p < 0.001). These results suggest that serum VEGF-165 may be helpful in therapy monitoring and surveillance of ovarian cancer patients and can provide additional information to CA-125 measurements. Given the high inter-study-variability of VEGF serum levels, a longitudinal measurement of individual patient levels might be a better approach than the assessment of absolute values at single time-points.

The prognostic potential of VEGF-165 levels after chemotherapy has also been described in a study evaluating the role of serum VEGF-165 in patients with complete response after first-line therapy [41]. These patients underwent second look surgery and women with persistent disease had higher VEGF serum levels. Taken together, these findings might add to the rationale of anti-VEGF therapy after completion of surgical and cytostatic first-line therapy of ovarian cancer, a concept currently under investigation in large phase III trials [42].

In contrast to previous findings by Li et al. [34] and Oehler et al. [36] we observed an initial postoperative increase of VEGF-165 and consecutive decrease during and after chemotherapy. The large wound area and healing process after radical debulking surgery often including extensive peritonectomy could explain the increase of angiogenic factors, as previously described for other malignancies [43]. Another reason for this difference might be the delayed collection of blood samples in the other series. Oehler et al. collected samples 4 weeks after surgery, a time at which most healing processes are already completed whereas in our study postoperative serum was collected within the first 7 days after surgery.

Matrix Metalloproteinases (MMPs) play a key role in invasion and metastasis of cancer cells. They are able to degrade extracellular matrix and aid angiogenesis [44]. MMPs can be regulated by their inhibitors, the tissue inhibitor of metalloproteinases (TIMPs) [45]. In addition to this regulatory role, TIMPs possess pro-tumorigenic and metastatic activity [46]. Increased expression of TIMP-1 has been associated with unfavourable outcome in several tumor types [47,48]. For ovarian cancer, conflicting results have been reported showing both up- and downregulation of TIMP-1 [49-51]. Rauvala et al. studied the level of TIMP-1 in preoperative serum of patients with ovarian cancer [52]. They found an elevated preoperative serum TIMP-1 to be associated with unfavourable clinical outcome, as previously suggested in a smaller study by Manenti et al. [40]. A negative prognostic impact of serum TIMP-1 has also been observed in breast cancer, colorectal cancer and other malignancies [53,54]. In the current study, we observed no correlation between pre- or postoperative serum concentrations of TIMP-1 and outcome (Table 3). However, low serum concentration at the end of chemotherapy was associated with improved overall survival (p = 0.007). Of note, TIMP-1 serum levels were closely correlated with VEGF-165 at all time-points (Figure 1 and 2). This could reflect common regulatory mechanisms and confirm the biological connection of both factors in ovarian cancer, leading to degradation of basement membranes and extracellular matrix as well as neovascularization.

In contrast to CA-125, a "normal value" could not be defined for TIMP-1 and VEGF-165 and different cut-off values have been used in previous studies [18,19,25,27-41,47,53-56]. We therefore assume that a longitudinal measurement of individual patient levels - using each patient as their own control - might be a better approach than the assessment of absolute values at single time-points.

Limitations of our study are the relatively small number of patients, its mono-centric design and the fact that samples were not strictly collected in consecutive patients, leading to a possible selection bias. However, the uniform treatment and high rate of patients with optimal surgical cytoreduction followed by platinum based combination chemotherapy might be a strength of this study. Biological markers are more likely to be detectable in patients without residual tumor burden, the most important unfavourable prognostic factor.

## Conclusion

This study is the first comprehensive longitudinal analysis of serum VEGF-165 and TIMP-1 in patients with ovarian cancer. It demonstrates that these markers change substantially during first-line therapy, are not correlated with CA-125 and might have prognostic relevance, suggesting a potential role in the surveillance of women with ovarian cancer. To further understand this role, the evaluation of TIMP-1 and VEGF in the context of prospective clinical trials is highly desirable.

## Competing interests

ELISA kits were provided at no cost by Siemens Medical Solutions Diagnostics. One co-author (WC) is employee of Siemens Medical Solutions Diagnostics; the other authors declare that they have no competing interests.

## Authors' contributions

SM participated in the design and coordination of the study, drafted the manuscript and participated in the statistical analysis. LW participated in the design and coordination of the study, drafted the manuscript and participated in the statistical analysis. CE participated in the design of the study and performed the statistical analysis and helped to draft the manuscript. JS participated in the design and coordination of the study. WC participated in the design of the study and helped to draft the manuscript. FJ participated in the design and coordination of the study and interpretation of the results. KML carried out the immunoassays, helped to draft the manuscript and participated in the statistical analysis. VM participated in the design and coordination of the study, drafted the manuscript and participated in the interpretation of the results. All authors read and approved the final manuscript.

## Pre-publication history

The pre-publication history for this paper can be accessed here:

http://www.biomedcentral.com/1471-2407/10/139/prepub
